# Suppressing ZnO-Induced Decomposition in Perovskite Solar Cells via Glycine-Based Chelation Strategy

**DOI:** 10.1021/acsami.5c13686

**Published:** 2025-11-07

**Authors:** Jannatul Ferdous, Md. Emrul Kayesh, Mostafa F. Abdelbar, Wipakorn Jevasuwan, Ashraful Islam, Naoki Fukata

**Affiliations:** † Research Center for Materials Nanoarchitectonics (MANA), 52747 National Institute for Materials Science (NIMS), Namiki, Tsukuba, Ibaraki 305-0044, Japan; ‡ Photovoltaic Materials Group, Research Center for Energy and Environmental Materials (GREEN), National Institute for Materials Science (NIMS), Sengen, Tsukuba, Ibaraki 305-0047, Japan; § Graduate School of Pure and Applied Sciences, University of Tsukuba, Tennodai, Tsukuba, Ibaraki 305-8573, Japan; ∥ Institute of Nanoscience & Nanotechnology, Kafrelsheikh University, Kafrelsheikh 33516, Egypt; ⊥ Faculty of Pure and Applied Sciences, University of Tsukuba, Tsukuba, Ibaraki 305-8573, Japan

**Keywords:** perovskite solar cell, decomposition, ZnO passivation, chelating agent, GlyAcid, GlyHCl

## Abstract

Organic–inorganic perovskite solar cells (PSCs) are a promising technology in renewable energy due to their high efficiency and low cost. The electron transport layer (ETL) plays a very important role in improving device performance by minimizing recombination losses and selective electron contact. In solar cells, zinc oxide (ZnO) is the most favored ETL due to its visible transparency, suitable energy level, excellent electron mobility, and structural flexibility. Nevertheless, the current application of ZnO in PSCs is restricted by the undesirable reverse decomposition reaction at the ZnO/perovskite (PVK) interface. The presence of surface hydroxyl (−OH) groups and interstitial zinc ions (Zn^2+^) speeds up the decomposition process. This process deteriorates the charge-collecting efficiency and PSC stability. By presenting a simple yet efficient technique for passivating the ZnO surface with chelating agents, glycolic acid (GlyAcid) and glycine hydrochloride (GlyHCl), we successfully addressed PVK decomposition at the ZnO/PVK interface. These chelating agents effectively passivated the ZnO surface through the suppression of −OH groups and the formation of metal complexes with interstitial Zn^2+^. It is found that, in comparison to GlyHCl, GlyAcid passivates the ZnO more effectively to form thermally stable optimum PVK on it with improved charge extraction, reduced defect density, larger grain size, and better energy level alignment. As a result, the ZnO/GlyAcid-based PSCs achieved a power conversion efficiency (PCE) of 23.09%, which is the highest PCE among the reported ZnO-based PSCs. Our research establishes a pathway to prevent PVK degradation on ZnO, allowing us to utilize its desirable ETL properties for PSCs.

## Graphical abstract

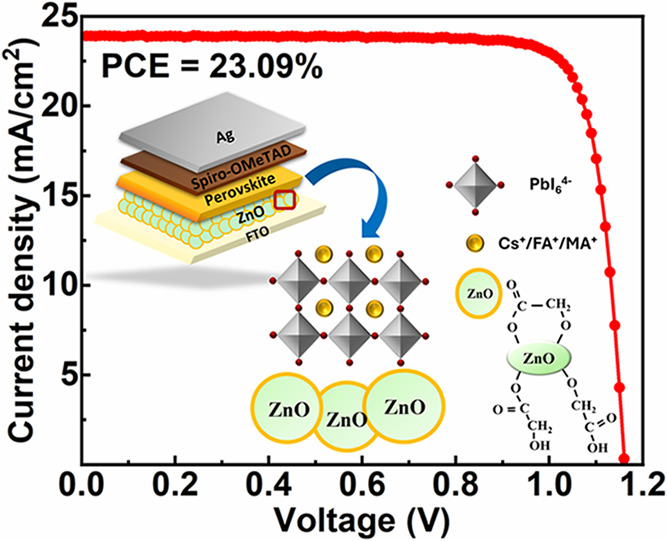

## 1 Introduction

Organic–inorganic hybrid perovskite solar cells (PSCs) have become a potential technology for the upcoming generation of solar cells over the last ten years.^1–3^ Along with their optimum optical characteristics, such as longer diffusion lengths of charge carriers, high molar extinction coefficients, adjustable band gaps, high mobility of charge carriers, etc., they also have simple-solution processing, low production costs, and high-power conversion efficiency (PCE).^4–7^ Perovskites (PVKs) are excellent materials for photovoltaic applications due to their outstanding characteristics. Among all thin-film photovoltaics, PSCs have the highest PCE, increasing from 3.8 to 26.95% since 2009.^8,9^ Apart from their high efficiency, PSCs’ instability as a result of their sensitivity to oxygen and moisture, as well as their reactivity with charge transport layers (CTL), has severely restricted their practical use.^2,7,10^

An electron transport layer (ETL) in a PSC contributes significantly to its efficiency and stability. ETL should be transparent in the visible spectrum, match the PVK material’s energy level, and exhibit high electron mobility to get excellent device output.^10^ Titanium dioxide (TiO_2_) and tin dioxide (SnO_2_) are typical ETLs in PSCs, realizing efficiency above 26%.^11,12^ At the PVK layer and CTL interface, efficient photogenerated carrier separation and extraction play important roles in efficient PSCs. The electron mobility of TiO_2_ and SnO_2_, on the other hand, is poor compared to the hole mobility of commonly used hole-transporting layers such as 2,2′,7,7′-tetrakis­[*N*,*N*-di­(4-methoxyphenyl)­amino]-9,9′-spirobifluorene (spiro-OMeTAD) and poly­[bis­(4-phenyl) (2,4,6-trimethylphenyl)­amine] (PTAA), which would lead to charge accumulation and deteriorate the functionality of the device. In this regard, ZnO can be a potential candidate because of its low-temperature processability,^7^ higher electron mobility (205–300 cm^2^v^–1^s^–1^),^13^ well-matched energy level to that of PVK materials, and high optical transmittance in the visible range.^14^ Many techniques, including atomic layer deposition (ALD), sol–gel, and RF sputtering, can be used to produce ZnO, which makes it simple to crystallize and dope to enhance its characteristics.^10^

Though ZnO has high potential, it is overlooked because the chemical instability of the ZnO/PVK contact causes the PVK to decompose quickly. Research indicates that as temperatures rise beyond 90 °C, the methylammonium cation in PVK thin films deprotonates, releasing methylamine and causing the PVK to decompose.^15,16^ The existence of surface hydroxyl (−OH) groups or leftover organic acetate ligands speeds up the breakdown process. The annealing of ZnO films leads to the disappearance of −OH and acetate groups, leaving only a small amount of acetate by 400 °C.^15^ According to Yang et al.,^15^ ZnO/MA­(MA = CH_3_NH_3_)­PbI_3_ contact experiences proton-transfer reactions due to the basic characteristics of the ZnO surface, which causes the PVK film to decompose. Thermal treatment of ZnO film at 400 °C increases the stability of the PVK layer; nevertheless, it also causes ZnO nanoparticles to aggregate, which slightly reduces PCE.^15^ Recently, Troshin et al. synthesized ZnO films with acetate, −OH, and amine-terminated groups and found that the −OH-terminated ZnO films were more reactive toward MAPbI_3_ and CsFA­(FA = CH_5_N_2_)­PbI_3_ perovskite.^17^ These problems are somewhat resolved by ALD, magnetron sputtering, and combustion synthesis techniques.^18^ Nevertheless, the production cost of PSCs is raised by these methods’ lengthy, complex, and costly facilities.^10^

In addition to these attempts, significant efforts have been undertaken to eradicate these types of interfacial reverse decomposition processes. Doping is an effective approach to modify the surface properties of ZnO, which can enhance the stability of PVK on ZnO while preserving the merits of sol–gel ZnO.^19,20^ Lin et al. claimed that the deprotonation reaction of MA cations is due to the higher basic nature of ZnO, and they weakened the basic properties by doping with Al and successfully prevented the deprotonation reaction.^21^ Miyasaka et al. used Mg as a dopant for ZnO ETL to form Zn–O–Mg, which retard the −OH group formation responsible for thermal instability.^22^ Alkali metals (LiOH, NaOH, and KOH) were used by Ahn et al. to dope ZnO, decreasing surface −OH groups, with increased stability, and produced a PCE of 19.90%.^23^ Another potential way to prevent the deprotonation reaction is to insert a passivation layer between ZnO and PVK. Besides surface modification, the utilization of the passivation layer can influence the morphology and crystallinity of the overlying PVK film needed for better optoelectronic properties. Although polyethylenimine^7^ and polyethylenimine ethoxylated^24^ were employed as ZnO and PVK interfacial modifiers, their insulating qualities may decrease charge transport and PSC’s performance.^25^

Research has been done on organic-molecule-based self-assembled monolayers (SAMs) on ETL as a substitute for polymer surface modification. To modify the ZnO film surface, Zuo et al. used a SAM of 3-aminopropionic acid (C3-SAM), which matched the energy level at the ZnO/PVK interface and created a permanent dipole moment.^26^ Numerous organic compounds have been investigated as surface modifiers for ETLs to create SAM layers, including JTCA,^27^ 3-(aminopropyl) triethoxysilane [APTES],^28^ graphene,^29^ 3,4,5-trimethoxybenzoic acid (TMBA),^30^ and ethanolamine (EA).^2^ However, the performance of SAM-modified ZnO-based PSCs still lags significantly behind that of existing PSCs. A chelating agent would be a better option to obtain the full advantages of sol–gel ZnO as an ETL. Chelating agents have a strong tendency to coordinate with metal ions and passivate the metal ions to prevent unwanted reactions. Recently, Yang et al. employed ethylene diamine tetraacetic acid (EDTA), a powerful chelating agent, as a dopant in ZnO solution to form EDTA-ZnO complexed ETL.^10^ In this case, EDTA nullifies the organic ligands on the ZnO surface that are responsible for PVK decomposition. However, the EDTA is a large chelating molecule, which may hamper the smooth charge transfer from PVK to ETL. Besides the −OH group, some researchers have reported the presence of interstitial zinc ions (Zn^2+^) on the surface of ZnO.^31–33^ These interstitial Zn^2+^ undergo a chemical reaction with perovskite to form nonphotoactive materials, thereby hindering device performance.^6^ Therefore, the potential advantages of smaller chelating molecular agents in selectively chelating the reactive sites of Zn^2+^ and organic molecules on ZnO, without hindering charge transfer, may be beneficial for utilizing the full advantages of ZnO as an ETL.

In this report, we used two small molecular chelating agents, glycolic acid (GlyAcid) and glycine hydrochloride (GlyHCl), to passivate the ZnO ETL by forming a coordination bond with Zn^2+^ ions. This makes it possible to form thermally stable PVK on ZnO at 150 °C. We found that the passivated ZnO contained a reduced amount of surface −OH groups, which are responsible for PVK decomposition. In addition, the passivated ZnO offered well-matched energy levels with PVK and an optimum surface on which PVK formed with high crystallinity, single-grain structure across the cross section, and optimal optoelectronic properties. Consequently, the passivated ZnO-based PSCs achieved a PCE of 23.09%, which is among the highest reported for ZnO-based PSCs. Interestingly, the unencapsulated ZnO/GlyAcid-based PSC maintained over 90% of its initial PCE for approximately 1800 h when stored in air at room temperature (23–25 °C) with a relative humidity of 40–50%. This study demonstrates that the passivation of ZnO by small molecular chelating agents is more appropriate for utilizing the full advantages of sol–gel ZnO as ETL for PSCs.

## 2 Experimental Section

### 2.1 Materials

Zinc acetate dihydrate (Zn­(CH_3_COO)_2_·2H_2_O) (ZnAc), 2-aminoethanol (H_2_NCH_2_CH_2_OH), 2-methoxyethanol (CH_3_OCH_2_CH_2_OH), GlyAcid (HOCH_2_COOH), GlyHCl (C_2_H_6_ClNO_2_), *N*,*N*-dimethylformamide (DMF), dimethyl sulfoxide (DMSO), ether, acetone, and ethanol were purchased from FUJIFILM Wako Pure Chemical Corporation. Formamidinium iodide (FAI, >98%), methylamine hydrobromide (MABr, 98%), lead­(II) bromide (PbBr_2_, 98%), lead­(II) iodide (PbI_2_, 99.99%), tris [2-(1*H*-pyrazol-1-yl)-4-tert butylpyridine]-cobalt- (III) tris­[bis­(trifluoromethylsulfonyl)­imide] (FK209, >99%), 4-*tert*-butylpyridine (4*-t*BP, >96%), and bis­(trifluoromethane) sulfonimide lithium salt (LiTFSI, >99%) were purchased from Tokyo Chemical Industry (TCI, Japan). 2,2′,7,7′-Tetrakis (*N*,*N*-di-*p*-methoxyphenylamine)-9,9′-spirobifluorene (Spiro-OMeTAD, >99.5%) was purchased from the Luminescence Technology Corporation, Taiwan. The cesium iodide (CsI, 99%), anhydrous chlorobenzene (CB, 99.5%), and isopropyl alcohol (IPA, ≥99.8%) were purchased from Sigma-Aldrich.

### 2.2 Synthesis of ZnO Film Passivated by GlyAcid and GlyHCl

The zinc sol–gel precursor solution was prepared by dissolving 250 mg of ZnAc and 64 μL of 2-aminoethanol into 3.75 mL of 2-methoxyethanol. The solution was stirred at 60 °C for 1 h, followed by stirring at room temperature for 24 h. The prepared solution was spin-coated onto the cleaned FTO substrate at 3000 rpm for 30 s and subsequently annealed at 150 °C for 20 min. It was subsequently annealed at 150 °C for 20 min. GlyHCl and GlyAcid (12.5 mg) were dissolved in 50 mL of deionized (DI) water and then stirred until they dissolved. The prepared ZnO substrates were separately dipped into solutions of GlyHCl and GlyAcid overnight. After being cleaned with DI water and dried using N_2_ gas, the ZnO substrates were spun for 50 s at 5000 rpm and then annealed at 100 °C for 5 min.

### 2.3 Synthesis of ZnAc GlyHCl Adduct

To synthesize the adduct of ZnAc and GlyHCl, a GlyHCl solution (2 mmol) was made by adding 150 mg of GlyHCl in 5 mL of DI water, and a ZnAc solution (1 mmol) was made by adding 220 mg of ZnAc in 2 mL of DI water. After that, the GlyHCl solution was added to the ZnAc solution. The mixed solution was then heated for 2 h at 100 °C while stirring. The solution was then concentrated using a rotary evaporator at low temperature. To precipitate the ZnAc GlyHCl adduct, 5 mL of acetone was added into the concentrated mixed solution. Finally, the white color adduct was collected and dried in a vacuum dryer for 2 h.

### 2.4 Synthesis of ZnAc GlyAcid Adduct

To synthesize the adduct of ZnAc and GlyAcid, a GlyAcid solution (2 mmol) was made by adding 152 mg of GlyAcid in 5 mL of DI water, and a ZnAc solution (1 mmol) was made by adding 220 mg of ZnAc in 2 mL of DI water. After that, the GlyAcid solution was added to the ZnAc solution. The mixed solution was then heated for 2 h at 100 °C while stirring. The solution was evaporated using rotary evaporation at a low temperature until white sediment was produced. Finally, the white adduct was washed with ether and dried in a vacuum dryer for 2 h.

### 2.5 Device Fabrication

FTO glass substrates with dimensions of 2.4 cm × 1.8 cm were patterned by etching with zinc powder and 2 M hydrochloric acid. The substrates were ultrasonically washed with detergent for 20 min and DI water, an IPA and ethanol mixture, IPA, and acetone for 20 min, respectively. After washing, the substrates were blown with N_2_ gas and placed in UV ozone for 30 min. Following that, the passivated ZnO films were moved into a glovebox that was filled with N_2_. A solution of perovskite (Cs_0.05_FA_0.80_MA_0.15_PbI_2.75_Br_0.25_) was made by dissolving CsI (0.1028 mmol), FAI (1.6454 mmol), MABr (0.3085 mmol), PbBr_2_ (0.1028 mmol), and PbI_2_ (2.0439 mmol) in a 3:1 DMF and DMSO mixed solvent and stirring for 1 h in a nitrogen (N_2_)-filled glovebox. The perovskite solution was spin-coated on the passivated ZnO film at a 1 s slope, 1000 rpm for 10 s, a 5 s slope, and 5000 rpm for 20 s. During this spin coating, CB was dripped onto the perovskite layer after 28 s and heated at 150 °C for 10 min. Spiro-OMeTAD was used as the HTL for the composition of FK209 (0.0018 mmol), LiTSFI (0.0299 mmol), spiro-OMeTAD (0.059 mmol), and 4*-t*BP (0.3184 mmol) and dissolved in 1 mL of CB. This solution was stirred in the glovebox for 30 min at 70 °C. After that, spiro-OMeTAD was deposited on the perovskite at a slope of 5 s, 4000 rpm for 20 s and a slope of 2 s. Finally, 100 nm of Ag was deposited by vacuum evaporation.

### 2.6 Characterization

The Shimadzu IRTracer-100 Fourier transform infrared (FTIR) spectrophotometer was utilized to measure the IR transmittance spectra. The JEOL ECS 400 NMR spectrometer, running at 400 MHz, was used to get proton nuclear magnetic resonance (^1^H NMR) spectra of passivated ZnO films. The X-ray photoelectron spectroscopy (XPS) and ultraviolet photoemission spectroscopy (UPS) measurements were carried out in a PHI VersaProbe 4 instrument (ULVAC PHI). A Hitachi S-8000 field-emission scanning electron microscope (FESEM) was used to view the surface morphology of passivated ZnO films and cross-sectional views of their devices. The atomic force microscopy (AFM) analysis was conducted in a Hitachi AFM-5000. ImageJ software was used to analyze the SEM images and calculate the perovskite films’ grain size. The overall average grain size and its distribution were determined by analyzing a large number of grains. The XRD pattern of the passivated ZnO films was obtained by using a MiniFlex 600 (Rigaku Co., Japan) powder X-ray diffractometer. The optical absorbance measurements were performed by using a Shimadzu UV–visible 3600 spectrophotometer. The PL and TRPL spectra were conducted in the Hamamatsu C12132 fluorescence lifetime spectrometer that used a 1.5 ns pulsed laser (frequency 15 kHz) with an excitation wavelength of λ = 532 nm and an excitation power of 0.1 mW. Under ambient conditions, the current–voltage (*J–V*) curves were measured using a solar simulator, an AM 1.5 G solar simulator (WXS-155S-10 from Wacom Denso Co., Japan). All devices’ *J–V* curves were measured by covering the cells with a metal mask for an active area of 0.09 cm^2^. The incident photon-to-current conversion efficiency (IPCE) analysis was done in a CEP-2000BX, Bunkoukeiki Co., Ltd. After the manufactured PSCs’ stability (shelf life) was assessed in an ambient setting, they were placed in a glovebox filled with N_2_. The Nyquist plot, transient photocurrent (TPC), and transient photovoltaic (TPV) decay were determined by the PAIOS (photo-analysis of impedance and optoelectronic signals) apparatus.

## 3 Results and Discussion

In this report, we selected GlyAcid and GlyHCl as small molecular chelating agents to neutralize Zn^2+^ and lower the −OH levels on the surface of ZnO. GlyAcid and GlyHCl are both classified as bidentate chelating agents because they each possess two coordination groups. They have a strong tendency to interact with Zn^2+^ through different functional groups and form stable complexes. GlyAcid has two functional groups: a carboxyl functional group (−COOH) and an −OH group. When ZnO is dipped into a diluted solution of GlyAcid, GlyAcid (C_2_H_4_O_3_) acts as a chelating agent by sticking to Zn^2+^ and forming a zinc-glycolate (Zn­(C_2_H_4_O_3_)_2_) complex. A stable five-membered ring forms around each Zn^2+^ ion when the oxygen atoms from the −COOH and −OH groups in GlyAcid attach to the Zn^2+^ on the surface of ZnO (Figure 1a).^34^ Besides this, both −COOH and −OH can donate a proton to the ZnO surface, breaking the bond between ZnO and the −OH. The newly formed −COOH and −OH will ionically bond with ZnO to help eliminate the −OH groups (Figure 1a).^35^ Meanwhile, the other chelating agent, GlyHCl, also has two functional groups: a −COOH and an amine (−NH_2_). In this case, glycine can bind to Zn^2+^ ions through both the –NH_2_ and −COOH groups, forming zinc-glycine (Zn­(C_2_H_5_NO_2_)_2_) complexes. These two functional groups allow glycine to interact with Zn^2+^, stabilizing it by encircling it with a five-membered ring structure (Figure S1).^36^ However, GlyHCl is less effective in reducing −OH from the surface of ZnO because of the presence of the –NH_2_ group. Typically, the –NH_2_ group does not participate in the reaction that breaks the ZnO–OH bond; rather, it inhibits the −COOH group due to the hindrance effect from donating a proton to the ZnO surface, which is necessary for breaking the bond between ZnO and the −OH (Figure S1).^37^

**Figure 1:**
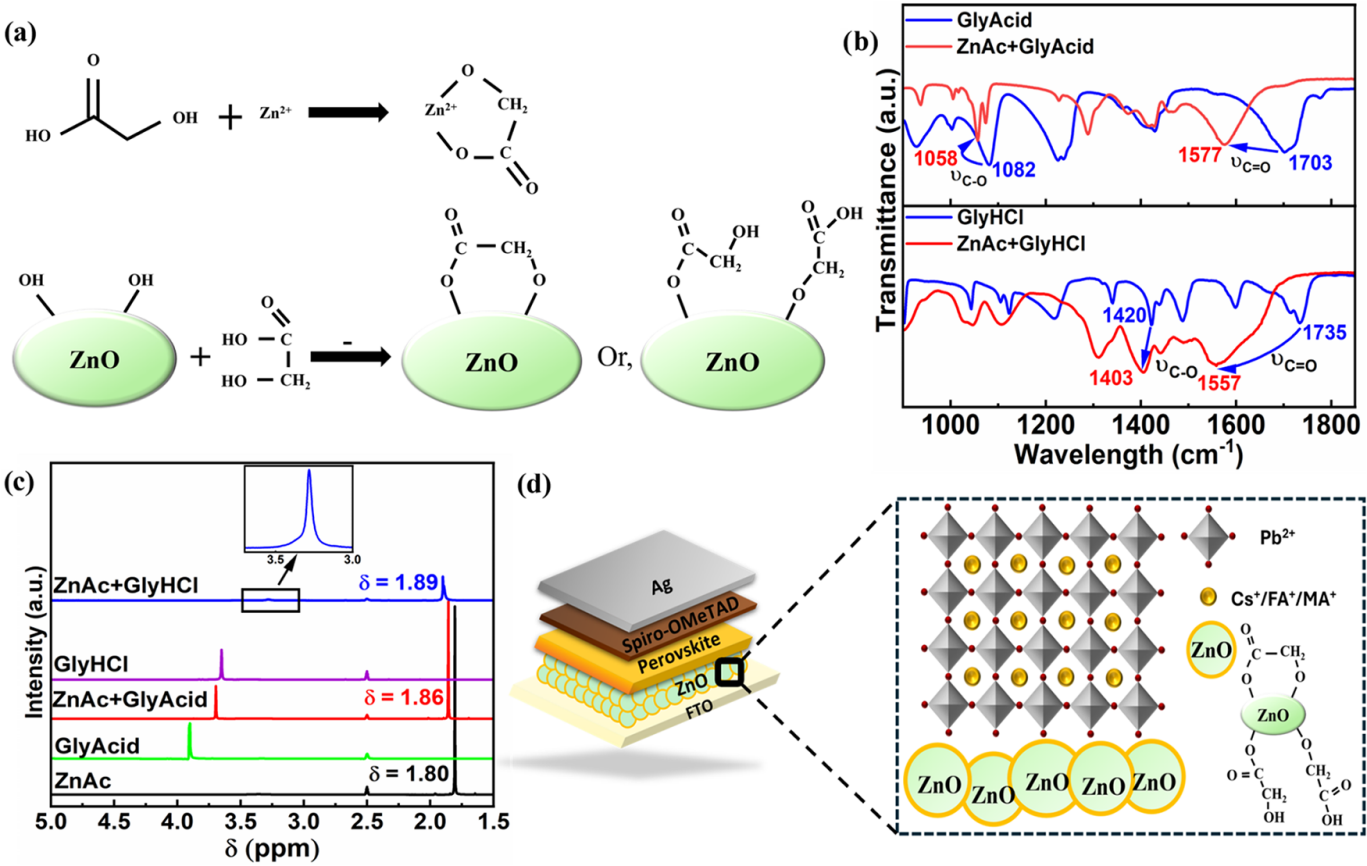
(a) Probable reaction mechanism occurring during the surface modification of ZnO with GlyAcid. (b) FTIR spectra of GlyHCl, ZnAc + GlyHCl, GlyAcid, and ZnAc + GlyAcid. (c)^1^H NMR spectra of ZnAc, GlyHCl, ZnAc + GlyHCl, GlyAcid, and ZnAc + GlyAcid in DMSO-*d*_6_ solution. (d) Schematic representation of the n-i-p Pb-PSC structure with an enlarged illustration showing how GlyAcid interacts with ZnO ETLs.

To observe the chemical interaction between ZnAc and GlyHCl and between ZnAc and GlyAcid, FTIR spectra were measured (Figure 1b). The distinctive FTIR peaks of GlyHCl at 1735 and 1420 cm^–1^ correspond to C=O and C–O stretching vibrations, respectively. Adduct formation using ZnAc causes the C–O band to move to a lower wavenumber at 1403 cm^–1^, while the 1735 cm^–1^ band vanishes and a new band arises at 1557 cm^–1^ for the C=O stretching bond.^38^ GlyAcid also shows peaks at 1703 cm^–1^ (C=O stretching) and 1082 cm^–1^ (C–O stretching) in its free molecule.^39^ In the ZnAc + GlyAcid adduct, the band at 1703 cm^–1^ disappears, giving rise to a new peak at 1577 cm^–1^, while the C–O band shifts to 1058 cm^–1^.^40^ The decrease of the C–O stretching vibrations (to 1403 and 1058 cm^–1^, respectively), the appearance of new peaks at 1557 and 1577 cm^–1^, and the disappearance of the original carbonyl peaks in both ZnAc + GlyHCl and ZnAc + GlyAcid adducts offer compelling evidence of complex formation between Zn^2+^ and the ligand molecules.

To validate the coordination among GlyAcid, GlyHCl, and Zn^2+^, we performed liquid-state ^1^H NMR measurements for GlyAcid, GlyHCl, and ZnAc solution, as shown in Figure 1c. The proton resonance peaks that appeared in ZnAc, GlyHCl, ZnAc + GlyHCl, GlyAcid, and ZnAc + GlyAcid solutions are assigned in Figures S2 and S3. From the ^1^H NMR spectra, we observed that the proton resonance peak of acetate from ZnAc appeared at 1.8 ppm. The proton resonance peak of acetate at 1.80 ppm shifted to 1.86 and 1.89 ppm with the addition of GlyAcid and GlyHCl in ZnAc solution. This indicates that GlyAcid and GlyHCl are effectively coordinated with ZnAc. Moreover, the peak intensity of acetate was reduced by up to 2 and 4 times when it interacted with GlyAcid and GlyHCl, respectively. These reductions in peak intensity are due to the slowing down of motion in the acetate molecule within ZnAc, indicating strong coordination with GlyAcid and GlyHCl.^41^ Moreover, the proton resonance peaks of GlyHCl and GlyAcid at 3.65 and 3.90 ppm are for −CH_2_–, respectively. After combination with ZnAc, these two peaks shifted to 3.35 and 3.69 ppm, respectively. It is noteworthy that combining ZnAc resulted in a greater decrease in the proton resonance peak intensity of −CH_2_– for GlyHCl compared to GlyAcid. This may be due to the coordination ability of GlyHCl being higher than that of GlyAcid. This observation is also supported by the FTIR results, where the ZnAc + GlyHCl adduct exhibited a greater shift in the C=O bond compared to the ZnAc+GlyAcid adduct (Figure 1b). Figure 1d represents an expanded picture of the n-i-p (where n is the ETL, i is the PVK layer, and p is the hole transport layer (HTL)) Pb-PSC structure showing how GlyAcid interacts with ZnO ETLs.

The presence of surface −OH groups on ZnO accelerates the PVK decomposition process of the PVK film deposited on ZnO.^24^ Therefore, the −OH groups need to be reduced for stable PVK. The impact of these chelating agents on deactivating the −OH groups present on the ZnO films was assessed by using X-ray photoelectron spectroscopy (XPS) measurements. A primary core level binding energy (BE) of Zn2P_3/2_ is visible in the XPS spectra at 1022 eV. The XPS peaks of Zn2P_3/2_ can be deconvoluted into two components by fitting with the Gaussian function located at 1020.93 and 1020.58 eV.^42^ ZnO is represented by the peak with a lower binding energy (1020.58 eV), while Zn­(OH)_2_ is responsible for the other peak (1020.93 eV). The passivation of GlyHCl and GlyAcid on ZnO causes the −OH percentages in the Zn­(OH)_2_ to drop from 31.62 to 17.05% and 6.72%, respectively (Figure 2a–c). To observe the aging effect, we exposed passivated ZnO in air for 3 h and measured XPS for Zn2P_3/2_. After exposing the passivated ZnO to air, we observed an increase in the proportion of −OH groups on both passivated ZnO samples, but the amount of −OH groups was significantly lower for ZnO/GlyAcid than for ZnO/GlyHCl (Figure S4). Besides the −OH group, the amphoteric ZnO surface abstracts H^+^ ions from organic cations (MA from PVK precursor), which triggers the degradation process of PVK.^43^ So, the reduction of −OH groups on the ZnO surface is not enough to stop the PVK degradation; effective passivation of the amphoteric ZnO surface is also needed for a stable PVK film on ZnO. The FTIR and ^1^H NMR results indicate that GlyHCl and GlyAcid effectively coordinate with ZnAc, providing direct evidence of their coordination ability with interstitial Zn^2+^ on the ZnO surface. After the surface modification of ZnO with GlyHCl and GlyAcid, the pristine ZnO’s root-mean-square (RMS) value dropped from 40.47 nm to 30.74 and 30.26 nm, respectively (Figure S5). This indicates that the −COOH and −OH groups were successfully anchored to the ZnO surface, resulting in a smoother surface that facilitates better contact and needs improved PSC performance.

**Figure 2:**
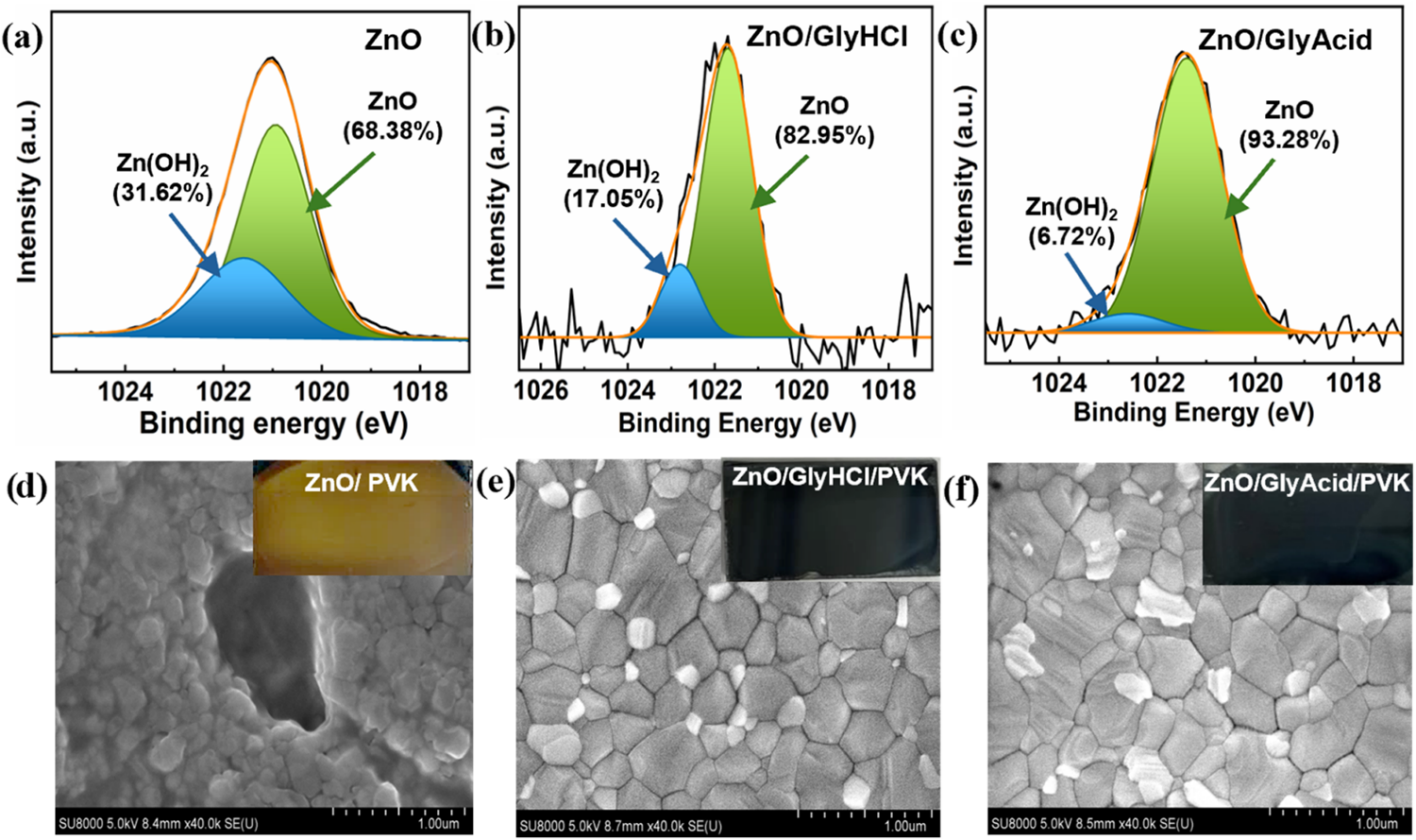
XPS deconvolution spectra for (a) pristine ZnO film, (b) ZnO/GlyHCl film, and (c) ZnO/GlyAcid film. SEM images (with insets showing the optical images of respective films) of PVK films deposited on (d) Pristine ZnO, (e) ZnO/GlyHCl, and (f) ZnO/GlyAcid.

Figure 2d–f displays scanning electron microscopy (SEM) images of PVK films deposited on ZnO, ZnO/GlyHCl, and ZnO/GlyAcid films. The PVK film deposited on ZnO initially formed a black film (the characteristic color of PVK), but when it was annealed at 150 °C, the PVK film started to degrade into a yellow color (the characteristic color of PbI_2_) (Figure 2d).^40^ The optical images of PVK films on ZnO, ZnO/GlyHCl, and ZnO/GlyAcid at different annealing (at 150 °C) time intervals show changes in color over time (Figure S6). As the PVK decomposes on the ZnO, the film’s morphology differs from the characteristics of the PVK grain structure. The degradation of the PVK films has been found to be accelerated by −OH groups and/or residual acetate ligands on the ZnO surface.^44^ On the other hand, the PVK film on ZnO/GlyHCl and ZnO/GlyAcid forms a compact and fully covered, pinhole-free PVK, maintaining its characteristic black color without degradation. This may be due to the reduction of −OH groups on the ZnO surface by the chelating agents GlyHCl and GlyAcid, as observed from the XPS results. Besides the reduction of −OH on the ZnO surface, the chelating agents such as GlyHCl and GlyAcid are firmly coordinated with interstitial Zn^2+^ to passivate it, which is needed to stop the degradation of PVK completely.

The passivated ZnO interacts with the PVK precursor solution and modifies the nucleation and growth dynamics of the PVK crystal. This modification can lead to a slower and more controlled crystallization process, resulting in improved film uniformity and stability. All films exhibit typical PVK morphologies when deposited on ZnO/GlyAcid and ZnO/GlyHCl, as shown in Figure 2e,f. Additionally, the films formed on ZnO/GlyAcid have typical grain sizes of 500 nm, which are significantly larger than those on ZnO/GlyHCl (352 nm). Figure S7 displays the grain size distribution diagrams. Although the chloride ions are known to have an influential effect on the growth of perovskite film, in this study, we did not find any appreciable change in the surface morphology of PVK film when comparing the two perovskite films deposited on GlyHCl and GlyAcid-passivated ZnO. This may be due to the insufficient chloride content on the GlyHCl-passivated ZnO’s surface. To evaluate the impact of ZnO surface passivation with GlyHCl and GlyAcid on the roughness of the perovskite film, AFM measurements were performed. As shown in Figure S8, the perovskite film on GlyAcid-passivated ZnO exhibited lower roughness as compared to that on GlyHCl-passivated ZnO. Furthermore, the PVK fabricated on ZnO/GlyAcid exhibited a greater water contact angle compared to PVK on ZnO/GlyHCl (Figure S9). This outcome signifies the increased hydrophobicity of PVK on ZnO/GlyAcid. This aligns with the SEM observations, as PVK on ZnO/GlyAcid exhibits a larger grain structure with fewer grain boundaries.

An X-ray diffraction (XRD) analysis was conducted to examine the crystallographic properties of the PVK deposited on both pristine and passivated ZnO, as depicted in Figure 3a. For pristine ZnO, we observed peaks only for PbI_2_, indicating the decomposition of PVK during annealing at 150 °C. This result is also consistent with the SEM observation (Figure 2d). We observed a yellow film on pristine ZnO, showing the characteristic color of PbI_2_. On the other hand, the XRD pattern of the PVK on passivated ZnO shows strong diffraction peaks at 14.1° and 28.3°, which can be attributed to the orthorhombic phase of the PVK’s (110) and (220) crystal planes.^45^ The chelating agents, GlyHCl and GlyAcid, passivate the ZnO layer, facilitating the growth of a more crystalline PVK phase without degradation. To evaluate the thermal stability of perovskite films deposited on ZnO/GlyHCl and ZnO/GlyAcid, XRD measurements were conducted before and after annealing at 70 °C for 300 h inside a glovebox (Figure S10). The perovskite on ZnO/GlyAcid retained its initial state, as evidenced by nearly unchanged PbI_2_ peak intensity. In contrast, the perovskite film on ZnO/GlyHCl exhibited a noticeable increase in the PbI_2_ peak intensity, indicating the partial degradation of its original phase. This difference can be attributed to the presence of a high density of −OH groups on the fresh ZnO/GlyHCl surface, as confirmed by XPS analysis (Figure 2b). These results suggested that the surface modification of ZnO by using GlyAcid is more effective than using GlyHCl to enhance the stability of the perovskite layer.

**Figure 3:**
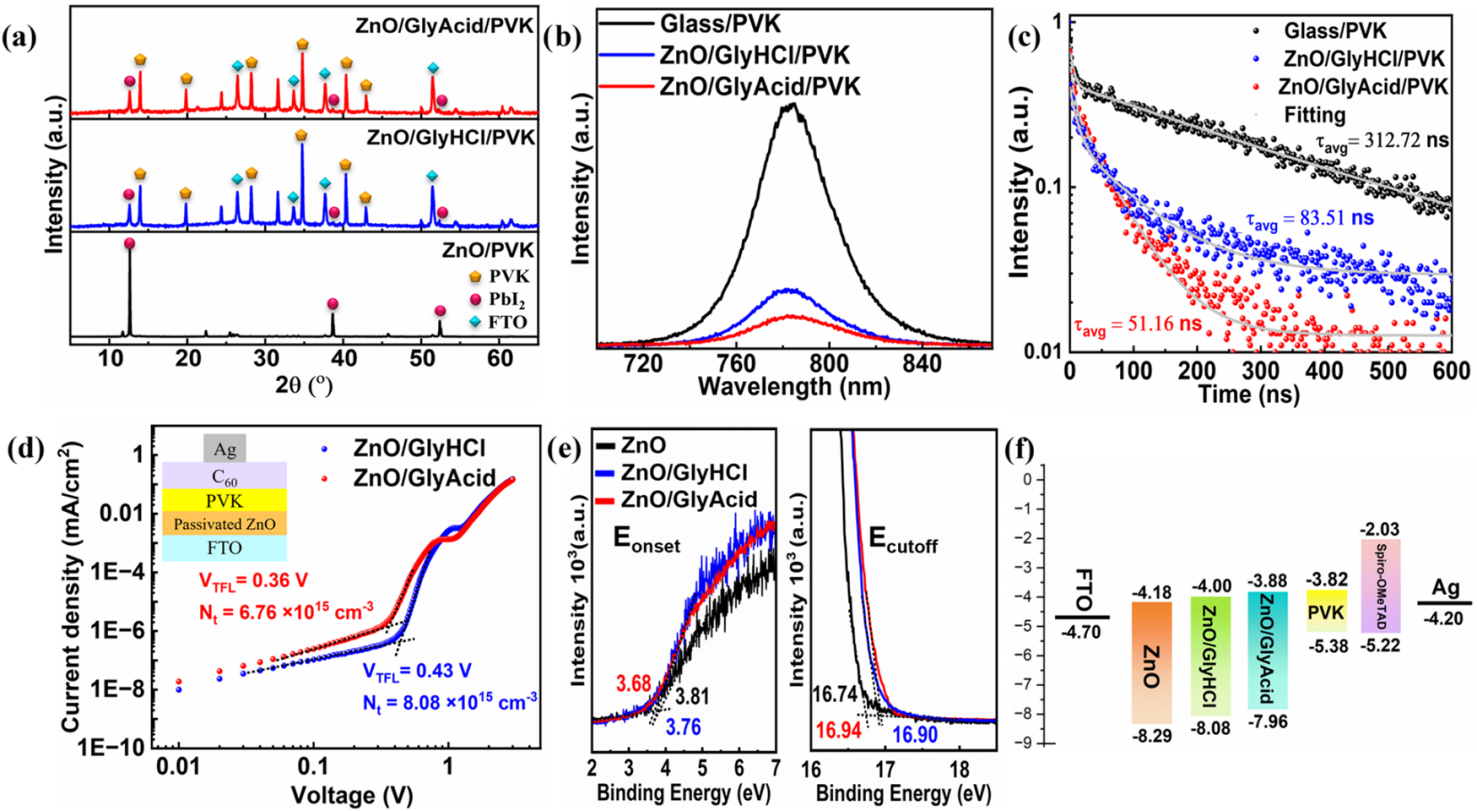
(a) XRD patterns of PVK films coated on pristine ZnO, ZnO/GlyHCl, and ZnO/GlyAcid. (b) Steady-state PL. (c) TRPL of PVK on glass, ZnO/GlyHCl, and ZnO/GlyAcid from the FTO substrate side. (d) SCLC measurements of ZnO/GlyHCl and ZnO/GlyAcid-based electron-only devices. (e) UPS spectra of ZnO, ZnO/GlyHCl, and ZnO/GlyAcid film. (f) Schematic illustration of energy diagrams of PVK, ZnO, ZnO/GlyHCl, and ZnO/GlyAcid films.

To investigate the transportation of photogenerated carriers from PVK to passivated ZnO, photoluminescence (PL) measurements were conducted (Figure 3b). We observed rigorous PL quenching in the steady-state PL for the PVK films deposited on passivated ZnO in comparison to PVK deposited on glass. This is due to the rapid charge transfer from the PVK to the passivated ZnO. The PL intensity of ZnO/GlyAcid/PVK is the lowest in comparison to ZnO/GlyHCl/PVK. According to this, ZnO/GlyAcid suppresses interface recombination and enhances electron transport at the ETL/PVK interface more effectively than ZnO/GlyHCl/PVK.^46^ We also performed time-resolved PL (TRPL) to gain a deeper understanding of the source of the PL quenching effect (Figure 3c). Trends in the steady-state PL and TRPL spectra are comparable. The TRPL curves were fitted using a biexponential decay function, which consists of a fast decay (τ_1_) and a slow decay (τ_2_) (eq S1). Table S1 also includes the corresponding fitting parameters. The calculated carrier lifetime (51.16 ns) for ZnO/GlyAcid/PVK is shorter than that of ZnO/GlyHCl/PVK (83.51 ns), as observed in the TRPL measurements. In this instance, it suggests effective electron transport at the interface, whereas a shorter TRPL lifetime often indicates faster recombination of charge carriers.^47^ Faster recombination in ZnO/GlyAcid/PVK is likely occurring at the interface, where enhanced electron transport prevents charge carriers from accumulating and prevents undesired recombination in the bulk of the PVK material. This is needed for an enhanced photovoltaic device performance.

Figure 3d shows typical dark current density vs voltage (*J–V*) curves for ZnO/GlyHCl- and ZnO/GlyAcid-based electron-only devices used to assess the defect density (*N*_t_) in PVK films deposited on passivated ZnO using the Space-Charge-Limited Current (SCLC) method. The trap-filling limit voltage (*V*_TFL_) was determined from the intersection of the trap-filling zone and the ohmic conduction zone. The Supporting Information describes the formula used to calculate defect density (eq S2). The results in Figure 3d indicate that the ZnO/GlyAcid-based device (0.36 V) has a *V*_TFL_ value lower than that of ZnO/GlyHCl (0.43 V). Therefore, the corresponding *N*_t_ values for the ZnO/GlyAcid- and ZnO/GlyHCl-based devices are 6.76 × 10^15^ and 8.08 × 10^15^ cm^–3^, respectively. These findings suggest that GlyAcid effectively passivates ZnO to form PVK with the optimum photovoltaic properties. This result is also consistent with the SEM, PL, and TRPL results. This property is crucial for PVK on ZnO to minimize loss during carrier transfer and improve the open circuit voltage (*V*_OC_) of the PSC.

The effects of passivation using chelating agents, GlyHCl and GlyAcid, on the energy levels of ZnO were evaluated through ultraviolet photoelectron spectroscopy (UPS) measurements (Figure 3e). The band gaps of ZnO/GlyAcid and ZnO/GlyHCl were both found to be 4.08 eV in accordance with the absorption spectrum’s Tauc plot (Figure S11a,b). ZnO/GlyHCl has an *E*_cutoff_ value of 16.90 eV, whereas ZnO/GlyAcid exhibits a slight shift to 16.94 eV. The work functions for ZnO, ZnO/GlyHCl, and ZnO/GlyAcid were determined to be −4.48, −4.32, and −4.28 eV, respectively. Consequently, ZnO, ZnO/GlyHCl, and ZnO/GlyAcid have valence band maximums (VBMs) of −8.29, −8.08, and −7.96 eV, respectively. As illustrated in Figure 3f, the ZnO/GlyAcid conduction band minimum (CBM) is −3.88 eV, which is somewhat higher than the CBM of ZnO/GlyHCl (−4.00 eV). This brings the ZnO/GlyAcid CBM much closer to the PVK conduction band at −3.82 eV, reducing energy loss in PVK devices. These variations in the CBM imply that ZnO/GlyAcid enables more effective charge transfer, which reduces recombination. This is needed to improve the *V*_OC_ in devices.^48^

After fabricating the stable PVK layer onto passivated ZnO, we proceeded to fabricate the PSC with the planar structure of FTO/passivated ZnO/PVK/spiro-OMeTAD/Ag. The cross-sectional SEM images of this configuration for ZnO/GlyHCl- and ZnO/GlyAcid-based devices are shown in Figure 4a,b, respectively. For the ZnO/GlyHCl-based device, the orientation of the grain is random across the cross section of the device. This causes a disturbance in electron flow through the device due to the presence of grain boundaries, which are regarded as recombination centers. ZnO/GlyAcid-based devices possess only vertically oriented grain structures, which facilitate electron flow without obstruction from grain boundaries (Figure 4b). This is necessary for efficient device performance. Figure 4c displays the best *J–V* curve of PSCs based on ZnO and ZnO passivated with GlyAcid and GlyHCl, measured in the reverse scan under one-sun conditions. The characteristics of photovoltaic (PV) parameters are summarized in Table 1. The bare ZnO-based device displayed a very low PCE of 0.45%, a *V*_OC_ of 0.13 V, a *J*_SC_ of 12.47 mA cm^–2^, and an FF of 27.97%. This extremely poor performance was due to the instability of perovskite on bare ZnO. Upon annealing the perovskite precursor layer on bare ZnO (a necessary step for perovskite formation), initially, it forms a black perovskite phase. But, during annealing, this phase starts to decompose, and within 5 min, it is completely converted to PbI_2_, as evidenced by the dominant PbI_2_ peaks observed in Figure 3a. The device with GlyAcid-passivated ZnO achieved the highest efficiency of 23.09% with a short circuit current density (*J*_SC_) of 23.94 mA cm^–2^, a *V*_OC_ of 1.16 V, and a fill factor (FF) of 83.14%. On the other hand, the optimum ZnO/GlyHCl-based device demonstrated performance with a PCE of 20.19%, *J*_SC_ of 22.69 mA cm^–2^, FF of 80.90%, and *V*_OC_ of 1.10 V. Furthermore, the hysteresis index (HI) is calculated using eq S3 to evaluate interfacial charge accumulation based on the PCE for both forward and reverse scans (Figure 4d,e). The HI decreases from 0.10 (ZnO/GlyHCl) to 0.04 (ZnO/GlyAcid), indicating that GlyAcid effectively passivates ZnO, resulting in a decrease in the level of *J–V* hysteresis.

**Table 1:** Performance Parameters of ZnO, ZnO/GlyHCl and ZnO/GlyAcid-Based PSCs

ETL	scan direction	*J*_SC_ (mA/cm^2^)	*V*_OC_ (V)	FF (%)	PCE (%)
ZnO	reverse	12.47	0.13	27.97	0.45
ZnO/GlyHCl	forward	22.74	1.07	74.51	18.13
	reverse	22.69	1.10	80.90	20.19
ZnO/GlyAcid	forward	23.91	1.15	81.04	22.28
	reverse	23.94	1.16	83.14	23.09

**Figure 4:**
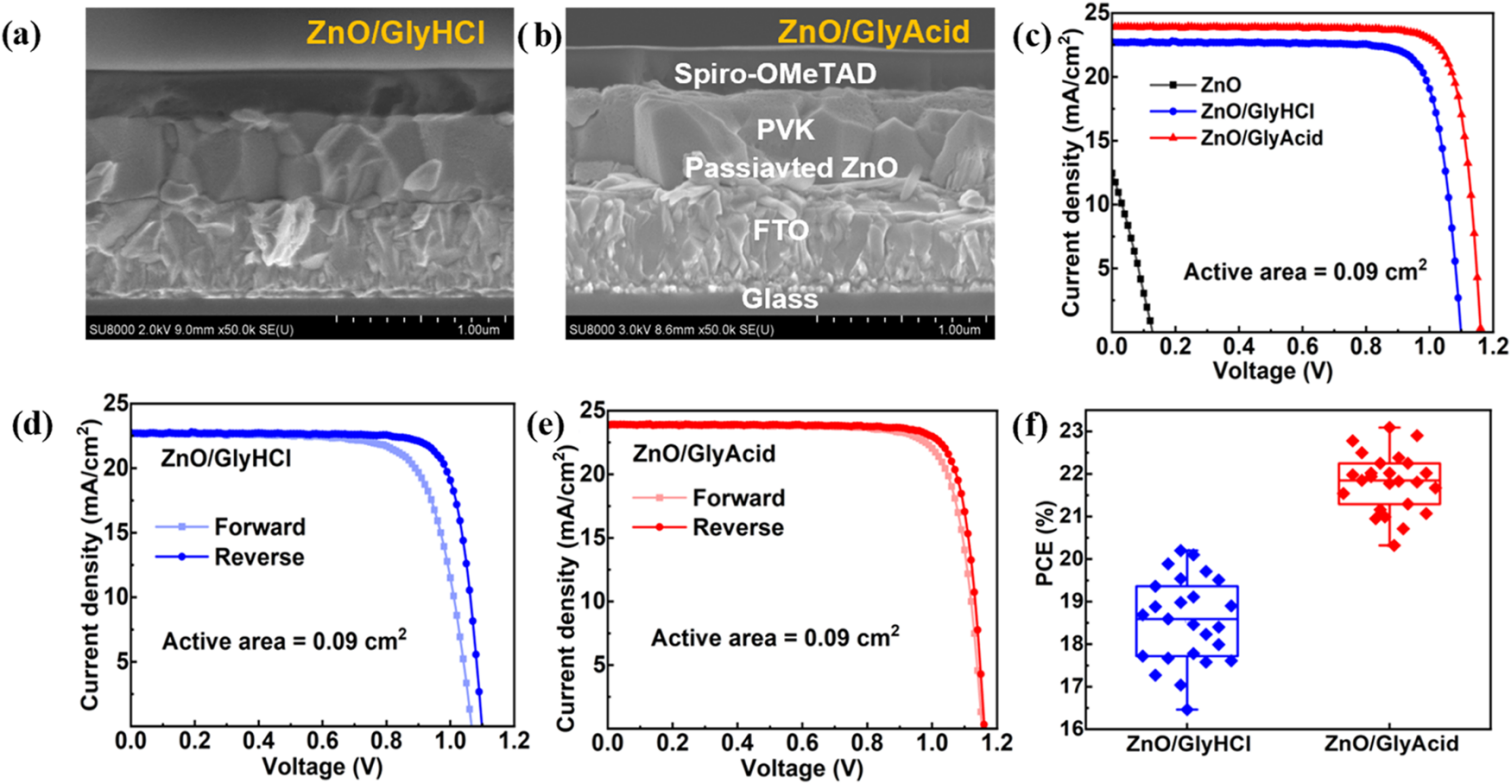
Cross-sectional SEM images of (a) ZnO/GlyHCl- and (b) ZnO/GlyAcid-based PSC. (c) *J*–*V* curve of ZnO, ZnO/GlyHCl, and ZnO/GlyAcid-based PSCs in reverse scan. *J*–*V* curve of (d) ZnO/GlyHCl and (e) ZnO/GlyAcid-based PSCs in both forward and reverse scans. (f) Statistical distribution of PCE.

The PV performances of the various ZnO-based PSC modifications are summarized in Table 2. In contrast to other treatments on ZnO-based PSCs, ZnO/GlyAcid-based PSCs attained the highest PCE of 23.09%. A total of 25 cells were enumerated for each batch. The ZnO/GlyAcid-based devices demonstrated markedly enhanced reproducibility compared to the ZnO/GlyHCl-based devices (Figure 4f). The enhancement in performance for the ZnO/GlyAcid-based devices was attributed to simultaneous improvements in *V*_OC_, *J*_SC_, and FF.

**Table 2:** Comparison of PV Performance Parameters of the Present Work with Those of Reported ZnO-Based PSCs

ETL modification	PVK	PCE (%)	*V*_OC_ (V)	*J*_SC_ (mA/cm^2^)	FF (%)	refs
ZnO/MAI passivation	CsFAPbI_3_ (MA free)	18.80	1.07	23.80	75.00	^6^
ZnO-Cl	CH_3_NH_3_PbI_3_	19.04	1.10	22.19	77.73	^50^
Combustion Synthesized ZnO	CsFAPbI_3_ (MA free)	19.84	1.08	24.67	74.49	^17^
ZnO/EDTA	MAPbI_3_	20.39	1.11	23.59	76.2	^10^
ZnO/MgO–EA^+^	CsFAMAPbBr_X_ I_3‑X_	21.08	1.12	23.86	78.91	^2^
ZnO	CsFAPbI_X_Br_3‑X_ (MA free)	21.10	1.20	22.50	78.01	^34^
ZnO/GlyHCl	CsFAMABr_X_I_3‑X_	20.19	1.10	22.69	80.90	present work
ZnO/GlyAcid		23.09	1.16	23.94	83.14	

To justify the *J*_SC_, we also measured incident photon-to-current efficiency (IPCE), and from the IPCE measurement (Figure 5a), we calculated short *J*_SC_ values of 23.50 and 22.23 mA cm^–2^ for GlyAcid and GlyHCl-passivated ZnO-based PSCs, respectively, which is consistent with the *J*_SC_ measurement from the *J–V* curve. As shown by the SCLC measurement for the electron-only device, the trap density decreases from 8.08 × 10^15^ (ZnO/GlyHCl/PVK) to 6.76 × 10^15^ cm^–3^ (ZnO/GlyAcid/PVK). This suggests that the passivation of ZnO by GlyAcid can reduce electron traps and enhance carrier transport, which may be a reason for improved *J*_SC_ and FF.^49^ This result aligns with the formation of single-grain PVK structures throughout the device cross section in ZnO/GlyAcid-based PSCs. On the other hand, the improved energy level alignment of ZnO/GlyAcid and PVK, as well as a decreased defect density of ZnO/GlyAcid/PVK film, could be the cause of the *V*_OC_ enhancement.

**Figure 5:**
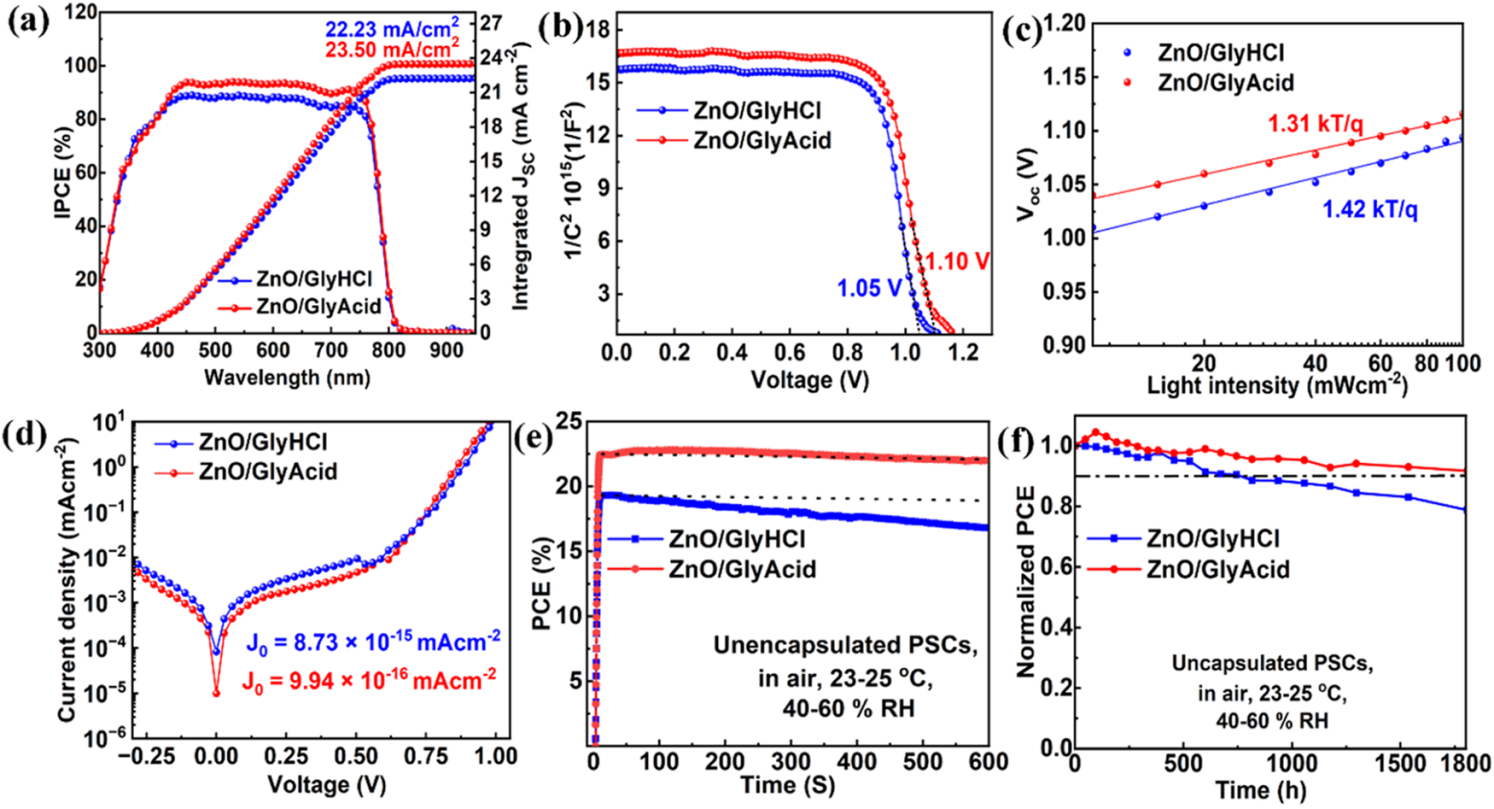
(a) IPCE spectra, (b) Mott–Schottky analysis, (c) *V*_OC_ vs incident light intensity plot, (d) dark *J–V*, (e) MPPT for 600 s, and (f) Normalized storage stability of the ZnO/GlyHCl- and ZnO/GlyAcid-based PSCs.

To justify the increase in *V*_OC_, we conducted a Mott–Schottky analysis of the PSC and investigated how the passivated ZnO layer affects charge transportation behavior. According to Figure 5b, the built-in potential (*V*_bi_) of the ZnO/GlyAcid device is 1.10 V, which is higher than that of the ZnO/GlyHCl device (1.05 V). This increased *V*_bi_ indicates improved charge transport, which prevents carrier recombination and increases the *V*_OC_.^51,52^ To gain a thorough understanding of trap-assisted recombination, particularly Shockley–Read–Hall (SRH) recombination, an experimental study was conducted to investigate how light intensity affects changes in *J*_SC_ and *V*_OC_ (Figure 5c). The slope of the curve was derived from curve fitting using the eq S4. The slope value is known as the ideality factor (n_id_), representing trap-assisted SRH recombination. We calculated the n_id_ value to be 1.42 and 1.31 for ZnO/GlyHCl- and ZnO/GlyAcid-based PSCs, respectively. A reduced n_id_ value indicates diminished SRH recombination, which can be attributed to defects. Moreover, there is an inverse relationship between *V*_OC_ and the dark current density (*J*_0_), indicating that an increase in *V*_OC_ requires a decrease in leakage current.

Figure 5d depicts the dark *J–V* associated with ZnO/GlyHCl- and ZnO/GlyAcid-based PSCs. An order-of-magnitude reduction in *J*_0_ was observed for ZnO/GlyAcid-based PSCs compared to ZnO/Gly/HCl-based PSCs. The decrease in leakage current resulted from the efficient passivation of ZnO and the growth of single-grain PVK structures across the device’s cross section by GlyAcid. The transient photovoltage (TPV) decay of PSCs utilizing ZnO/GlyHCl and ZnO/GlyAcid ETL materials was examined to gain a deeper understanding of the recombination kinetics. TPV measurements revealed that ZnO/GlyAcid-based PSCs had a delayed TPV decay (2.15 ms) compared to ZnO/GlyHCl-based PSCs (1.49 ms) (Figure S12). The delayed decay of TPV indicates a longer lifespan of charge carriers and a decreased rate of recombination in ZnO/GlyAcid-based PSCs.^53^ Furthermore, examination of the transient photocurrent (TPC) can provide information about the carrier transit mechanism in PSCs. According to Figure S13, the ZnO/GlyAcid-based PSCs exhibit faster carrier transit, as indicated by their faster photocurrent decay (1.19 μs) compared to the ZnO/GlyHCl-based PSCs (3.11 μs). The reduced defect density in PVK film on ZnO/GlyAcid increased *V*_bi_, which may have contributed to the improved carrier transport rate in ZnO/GlyAcid ETL-based PSCs. Figure S14 shows the Nyquist plot for ZnO/GlyHCl- and ZnO/GlyAcid-based PSCs. The Nyquist plot consists of two arcs: large arcs at low frequency and small arcs at higher frequency. These arcs were fitted with the equivalent circuit, as shown in the inset of Figure S14. The large arc is associated with recombination resistance (*R*_re_), which is caused by charge recombination at the interface between the PVK and ETL. ZnO/GlyAcid-based PSCs consist of a large arc as compared with ZnO/GlyHCl-based PSCs, implying a longer recombination time, aligned with the respective device performance. This may be due to the effective passivation of ZnO and the optimum growth of PVK film on the ZnO/GlyAcid surface. On the other hand, a smaller arc corresponding to charge transport resistance (*R*_ct_) at higher frequency was observed for ZnO/GlyAcid-based PSCs, indicating better n-type characteristics of the ZnO/GlyAcid ETL and better conduction band alignment between the PVK/ZnO/GlyAcid interface.^54^

Another crucial aspect of PSCs’ overall performance is their long-term stability. To evaluate photostability, we therefore measured the stabilized PCE at the maximum power point tracking (MPPT) condition (Figure 5e). After being exposed to constant light illumination for 600 s, the unencapsulated ZnO/GlyAcid-based device’s PCE drops from 22.65 to 21.95%, or 97% of the initial values. In contrast, the ZnO/GlyHCl-based device retains only 87% (19.27 to 16.67%) of its initial values. Furthermore, the long-term self-stability was evaluated by storing unencapsulated ZnO/GlyHCl- and ZnO/GlyAcid-based devices in ambient conditions (in air, at 24–26 °C and relative humidity (RH) of 40–50%). The findings demonstrate that the ZnO/GlyAcid-based PSCs sustain an initial PCE of over 90% (Figure 5f) for 1800 h of storage in the open air. Nonetheless, the ZnO/GlyHCl-based PSCs maintain an initial PCE of 80%. This is primarily due to the effective passivation of the ZnO surface by the chelating GlyAcid and the growth of a large-grain structure, and the hydrophobic nature of the PVK film on passivated ZnO. Moreover, the SEM and water contact angle results indicate that the GlyAcid-passivated ZnO surface helps to grow larger single-grain structures and hydrophobic PVK film. These beneficial impacts of ZnO passivation by GlyAcid assist the PVK film in achieving improved stability in the ambient environment.

## 4 Conclusion

This study demonstrated an effective surface passivation strategy using chelating agents (GlyAcid and GlyHCl) to prevent degradation of PVK at the ZnO ETL/PVK interface. GlyAcid and GlyHCl form a stable metal complex with interstitial Zn^2+^ and reduce −OH at the ZnO surface, which prevents the PVK degradation on ZnO. The passivation of ZnO by GlyAcid produces superior ZnO films with better energy level alignment with PVK and makes an optimum surface for the growth of single-grained structure across the cross section of PSCs. This enhances the electron extraction capacity and suppresses recombination losses. All of these improvements resulted in an increase in PCE up to 23.09% for the ZnO/GlyAcid device, signifying a major breakthrough in the use of ZnO as a reliable ETL in high-performance PSCs. This demonstrates how well the passivation process maximizes ZnO as an ETL, leading to significant improvements in the stability and efficiency of the PSCs.

## Supplementary Material



## Notes

Probable reaction mechanism, ^1^H NMR spectrum, XPS deconvolution spectra, AFM images, optical images, grain size distribution, contact angle, XRD spectra, UV–vis absorption spectra, TPV plots, TPC plots, Nyquist plots, TRPL lifetime calculation, defect density calculation, hysteresis Index calculation, and Shockley–Read–Hall (SRH) recombination (PDF)
