# Supplementary material for: Suppressing ZnO-Induced Decomposition in Perovskite Solar Cells via Glycine-Based Chelation Strategy
Source: ACS Appl Mater Interfaces. 2025 Nov 7;17(46):63342–52. doi: 10.1021/acsami.5c13686 (PMC12635964; doi:10.1021/acsami.5c13686)
Supplement: Supplementary file 1 [file am5c13686_si_001.pdf]

Supporting Information

Suppressing ZnO-Induced Decomposition in Perovskite Solar Cells via Glycine-Based Chelation Strategy

Jannatul Ferdous[1,2,3], Md. Emrul Kayesh[2], Mostafa F. Abdelbar[4], Wipakorn Jevasuwan[1], Ashraful Islam[2], Naoki Fukata[1,5*]*

[1]Research Center for Materials Nanoarchitectonics (MANA), National Institute for Materials Science (NIMS), Namiki, Tsukuba, Ibaraki 305-0044, Japan

[2]Photovoltaic Materials Group, Research Center for Energy and Environmental Materials (GREEN), National Institute for Materials Science (NIMS), Sengen, Tsukuba, Ibaraki 305-0047, Japan

[3]Graduate School of Pure and Applied Sciences, University of Tsukuba, Tennodai, Tsukuba, Ibaraki 305-8573, Japan

[4]Institute of Nanoscience & Nanotechnology, Kafrelsheikh University, Kafrelsheikh 33516, Egypt

[5]Faculty of Pure and Applied Sciences, University of Tsukuba, Tsukuba, Ibaraki 305-8573, Japan

[*]Corresponding authors:

Naoki Fukata: fukata.naoki@nims.go.jp

Ashraful ISLAM: islam.ashraful@nims.go.jp

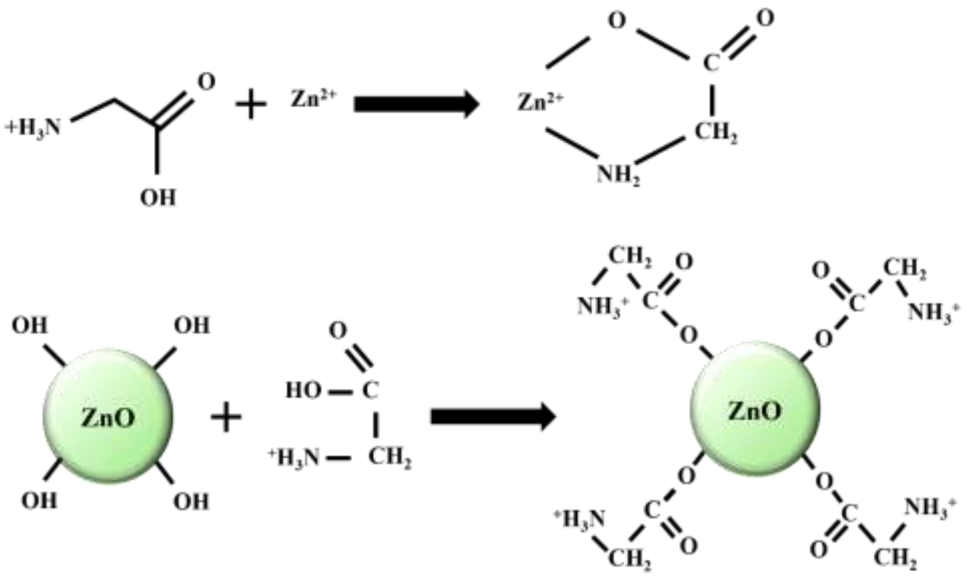

Figure S1. Probable reaction mechanism occurring during the surface modification of ZnO with GlyHCl.

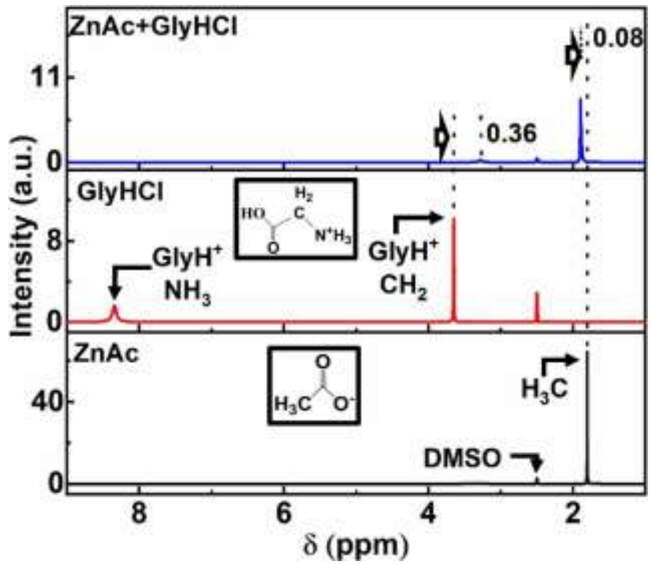

Figure S2. ^{1}H NMR spectrum of ZnAc (Zn $(CH_3CO_2)_2.2H_2O$), GlyHCl ($C_2H_6ClNO_2$), and ZnAc and GlyHCl mix solution in DMSO-d_6.

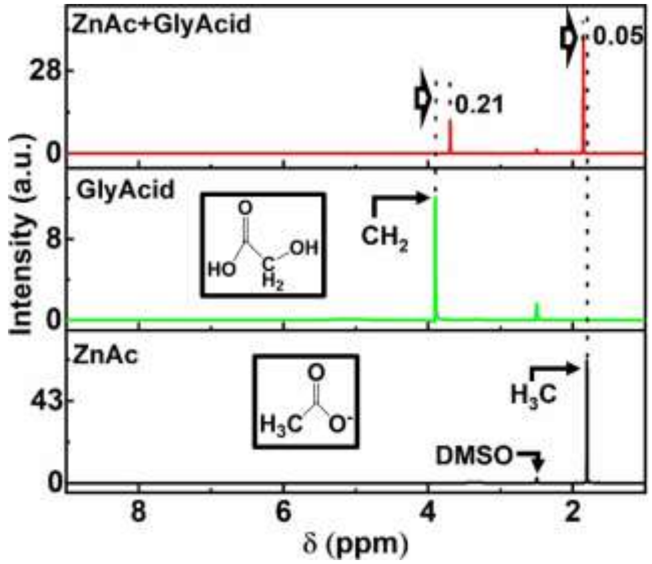

Figure S3. ^{1}H NMR spectrum of ZnAc (Zn $(CH_3CO_2)_2.2H_2O$), GlyAcid, ($C_2H_4O_3$), and ZnAc and GlyAcid mix solution in DMSO-d_6.

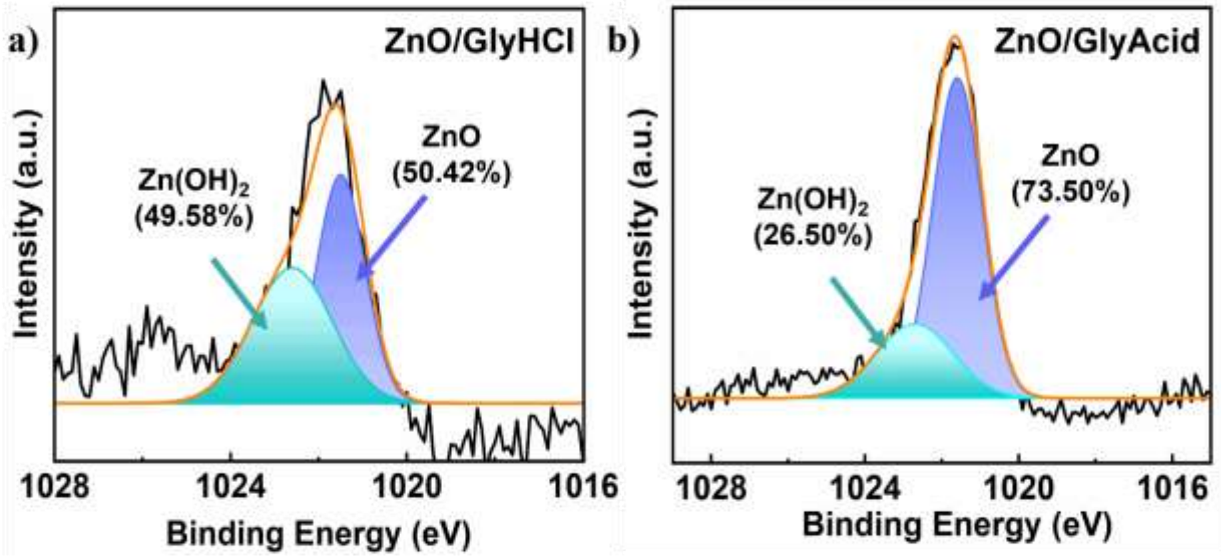

Figure S4. XPS deconvolution spectra for a) ZnO/GlyHC1 film, and b) ZnO/GlyAcid film in air at 25 °C and 75-85% RH.

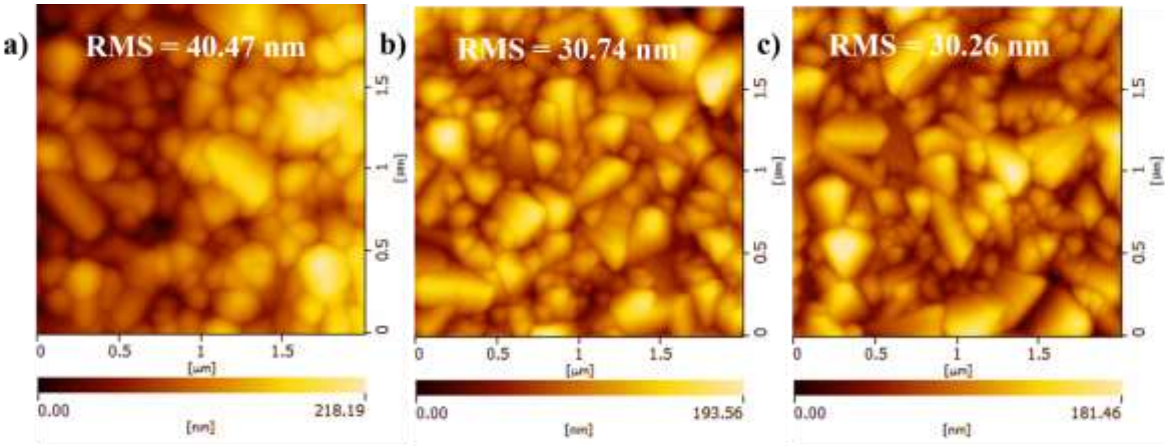

Figure S5. AFM topographical images of a) Pristine ZnO, b) ZnO/GlyHCl, and c) ZnO/GlyAcid films.

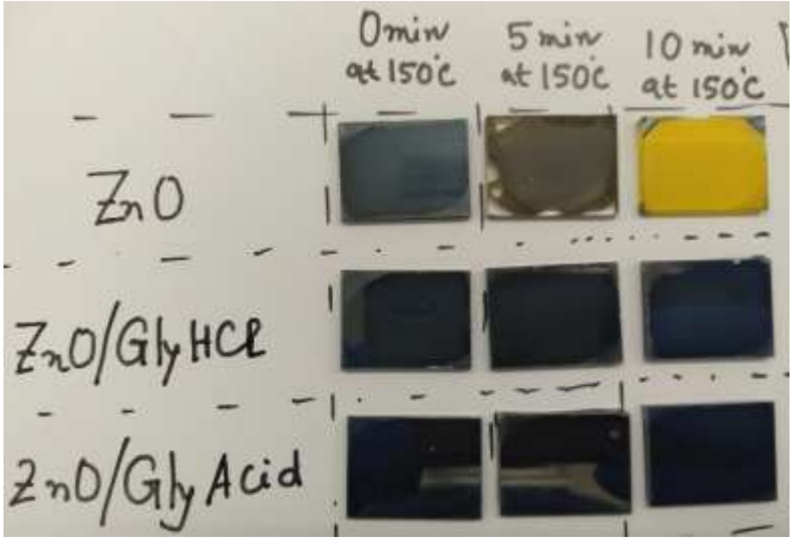

Figure S6. Optical images of Perovskite films on ZnO, ZnO/GlyHCl, and ZnO/GlyAcid at 150 °C for different annealing times. For effective passivation of ZnO by chelating agents like GlyHCl or GlyAcid, the immersion of ZnO substrate into GlyHCl or GlyAcid solution for at least 12 h is mandatory. If the immersion time becomes lower than 12 h, the perovskite on ZnO will decompose during the annealing (at 150 °C) due to incomplete passivation.

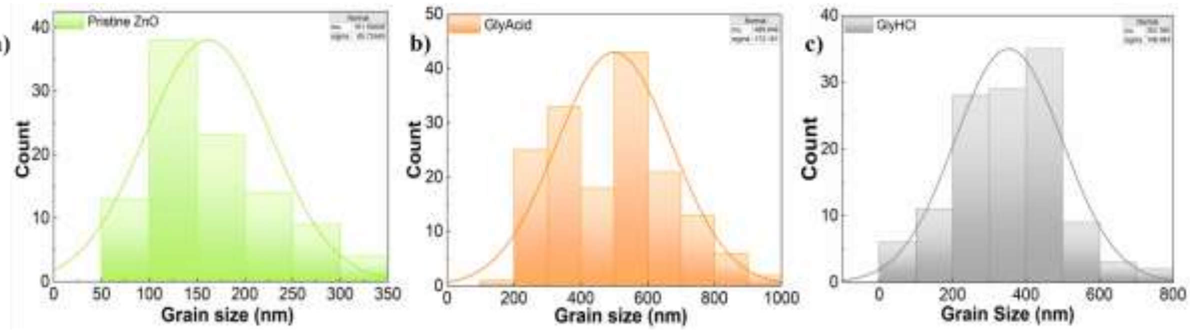

Figure S7. Grain size distribution of perovskite films on a) Pristine ZnO, b) ZnO/GlyAcid, and c) ZnO/GlyHCl.

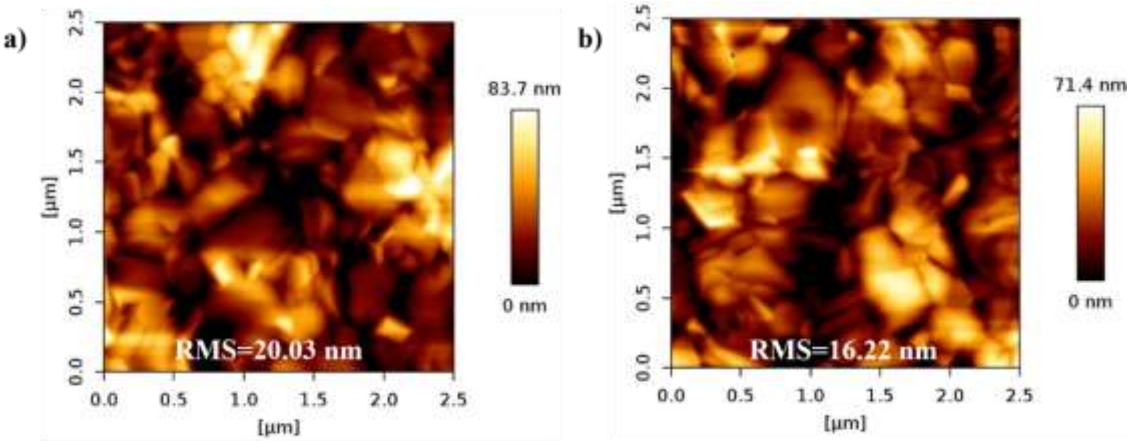

Figure S8. AFM topographical images of a) ZnO/GlyHCl/PVK and b) ZnO/GlyHCl/PVK films.

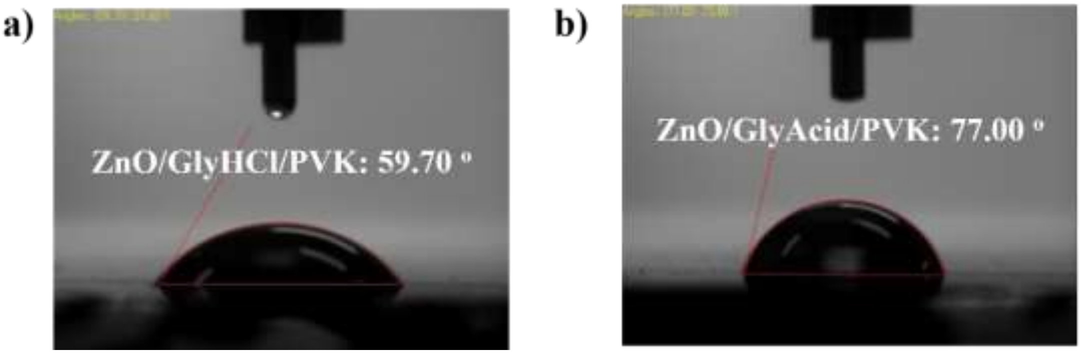

Figure S9. Water contact angle of a) ZnO/GlyHCl/PVK and b) ZnO/GlyAcid/PVK.

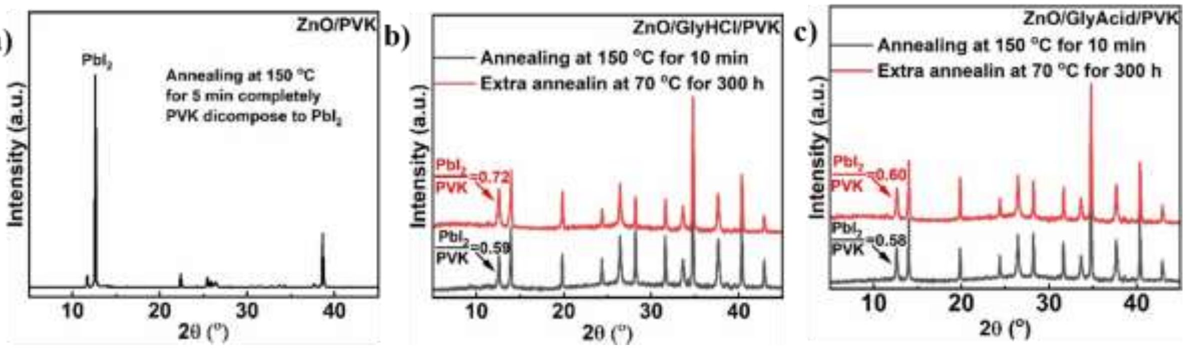

Figure S10. XRD spectra of perovskite films on a) ZnO, b) ZnO/GlyHCl, and c) ZnO/GlyAcid as a function of time at 70 °C. We have normalized the figures (a) and (b) with respect to 14 ° peak intensity to compare the change of PbI_2 peak intensity at 12.6 °.

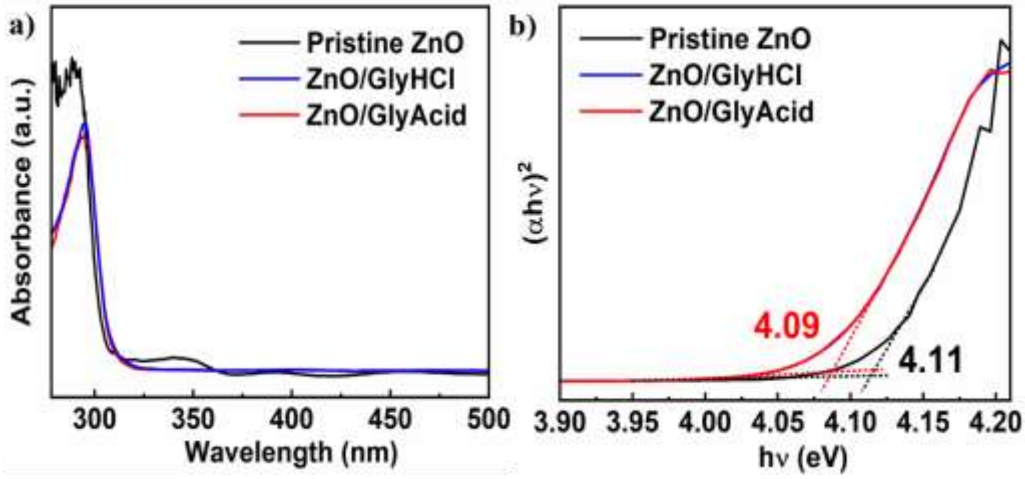

Figure S11. a) UV-vis absorption spectra and b) tauc plots of ZnO, ZnO/GlyAcid, and ZnO/ GlyHCl.

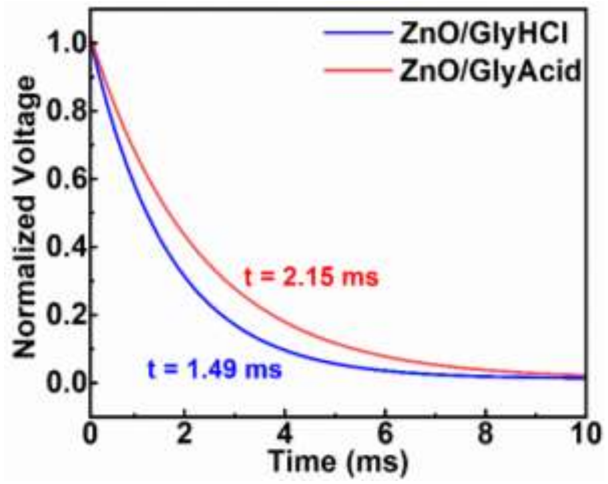

Figure S12. TPV plots of ZnO/GlyHCl- and ZnO/GlyAcid-based PSCs.

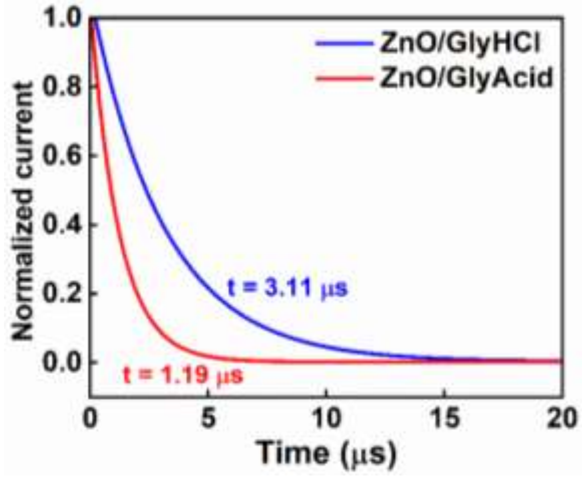

Figure S13. TPC plots of ZnO/GlyHCl- and ZnO/GlyAcid-based PSCs.

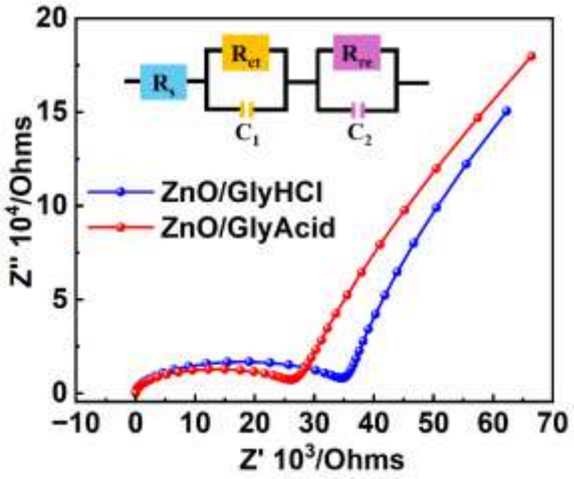

Figure S14. Nyquist plots of ZnO/GlyHCl- and ZnO/GlyAcid-based PSCs. The inset displays the equivalent circuit diagram.

TRPL lifetime calculation

The TRPL intensity decay curves are modeled using a bi-exponential decay function:

$$I(t) = I_0 + A_1 \exp(-t/\tau_1) + A_2 \exp(-t/\tau_2)$$

where, τ_1 and τ_2 represent fast and slow decay time constant, respectively.[1]

Average time constant (τ) is calculated using relation of,

$$\tau = \frac{A_1\tau_1^2 + A_2\tau_2^2}{A_1\tau_1 + A_2\tau_2)} \ldots\ldots\ldots\ldots\ldots\ldots\ldots\ldots\ldots\ldots S1$$

Defect density calculation

$$\text{Trap filling voltage, } V_{TFL} = \frac{qN_tL^2}{2\varepsilon\varepsilon_0} \ldots\ldots\ldots\ldots\ldots\ldots\ldots\ldots\ldots\ldots S2$$

where, q is the elementary charge of the electron = 1.6×10^{-19} C, L is the thickness of the perovskite film = 180 nm, ε is the relative dielectric constant of perovskite = 5.5, ε_0 is the dielectric constant of vacuum = 8.854×10^{-12} F·m^{-1}, V_{TFL} is the trap-filled limit voltage = 0.36 V (for ZnO/GlyAcid) and N_t is the defect density in cm^{-3}.[2]

Hysteresis Index calculation (HI):

$$HI = \frac{PCE_R - PCE_F}{PCE_R} \text{...S3}$$

Here, PCE_R = power conversion efficiency in the reverse scan, PCE_F = power conversion efficiency in the forward scan.

Shockley–Read–Hall (SRH) recombination:

The ideal factor (n_{id}) represents the trap-assisted SRH recombination, which can be computed using the following equation.[3]

$$V_{OC} = \frac{n_{id} k_B T}{q} \ln\left(\frac{J_{SC}}{J_0}\right) \text{..S4}$$

Here, q is the elementary charge, K_B is the Boltzmann constant, J_0 is the dark current density, V_{OC} is the open circuit voltage, and T is the absolute temperature.

Table S1. Fast and slow decay components extracted from the TRPL spectra.

ETLs	A_1 (%)	τ_1 (ns)	A_2 (%)	τ_2 (ns)	τ_{avg} (ns)
**Glass/PVK**	0.33	5.35	0.40	317	312.72
**ZnO/GlyHCl/PVK**	0.21	11.44	0.167	94.48	83.51
**ZnO/GlyAcid/PVK**	0.32	4.20	0.32	54.76	51.16

References:

1. Chen, J.; Kim, S.-G.; Ren, X.; Jung, H. S.; Park, N.-G. Effect of bidentate and tridentate additives on the photovoltaic performance and stability of perovskite solar cells. *J. Mater. Chem. A* **2019**, *7(9)*, 4977-4987. https://doi.org/10.1039/C8TA11977E.

2. Heo, J. H.; You, M. S.; Chang, M. H.; Yin, W.; Ahn, T. K.; Lee, S.-J.; Sung, S.-J.; Kim, D. H.; Im, S. H. Hysteresis-less mesoscopic $CH_3NH_3PbI_3$ perovskite hybrid solar cells by introduction of Li-treated TiO_2 electrode. *Nano Energy* **2015**, *15*, 530-539. https://doi.org/10.1016/j.nanoen.2015.05.014

3. Sun, X.; Shao, Z.; Li, Z.; Liu, D.; Gao, C.; Chen, C.; Zhang, B.; Hao, L.; Zhao, Q.; Li, Y.; Wang, X.; Lu, Y.; Wang, X.; Cui, G.; Pang, S. Highly efficient $CsPbI_3/Cs_{1-x}DMA_xPbI_3$ bulk heterojunction perovskite solar cell. *Joule* **2022**, *6(4)*, 850-860.
